# Cyclodextrin-Based Nanotransporters as a Versatile Tool to Manage Oxidative Stress-Induced Lung Diseases

**DOI:** 10.3390/antiox14081007

**Published:** 2025-08-17

**Authors:** Supandeep Singh Hallan, Francesca Ferrara, Maddalena Sguizzato, Rita Cortesi

**Affiliations:** 1 Department of Pharmaceutical Sciences and Natural Products, Central University of Punjab, Bathinda 151401, India; hllsnd@unife.it; 2 Department of Chemical, Pharmaceutical and Agricultural Sciences, University of Ferrara, I-44121 Ferrara, Italy; frrfnc3@unife.it (F.F.); sgzmdl@unife.it (M.S.); 3 Biotechnology Interuniversity Consortium (C.I.B.), Ferrara Section, University of Ferrara, I-44121 Ferrara, Italy

**Keywords:** oxidative stress, polyphenols, cyclodextrins, pulmonary drug delivery, inflammation, solubility, inclusion complex, lungs, drug delivery

## Abstract

Oxidative stress is one of the key elements in lung-related complications such as cystic fibrosis, acute lung injury, pulmonary hypertension, bronchopulmonary dysplasia, chronic airway diseases, lung cancer, COVID-19, and many others. Antioxidant and anti-inflammatory therapy can be considered as supportive alternatives in their management. However, most naturally derived antioxidants face issues with poor aqueous solubility and stability, which hinder their clinical utility. Remarkably, local pulmonary delivery circumvents the severe limitations of oral delivery, including hepatic first-pass metabolism and organ toxicity, and enables a higher drug payload in the lungs. Here, in this review, we present cyclodextrin as a potential drug carrier for pulmonary administration, exploring the possibilities of its surface modification, complexation with other drug transporters, and loading of cannabidiols, siRNA, and antibodies as future trends. However, the lack of a robust physiological model for assessing the efficacy of lung-oriented drug targeting is a significant concern in its path to clinical and commercial success.

## 1. Introduction

**Figure 1:**
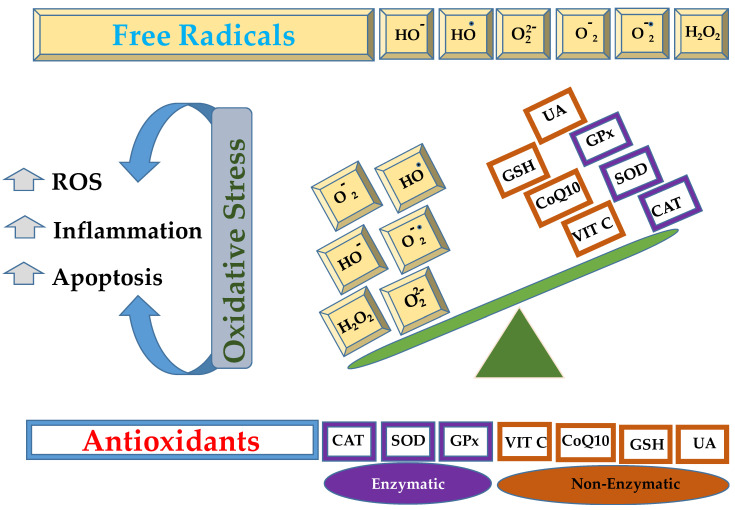
Schematic representation of imbalance between free radicals, such as hydroxide ion (HO^−^), hydroxyl radical (HO^•^), peroxide ion ($O_{2}^{2-}$), superoxide ($O_{2}^{-}$), superoxide anion ($O_{2}^{-•}$), hydrogen peroxide (H_2_O_2_), that can be neutralized by enzymatic (catalase, CAT; superoxide dismutase, SOD; glutathione peroxidase, GPx) or non enzymatic antioxidant mechanisms (vitamin C, VIT C; coenzyme Q10, CoQ10; reduced glutathione, GSH; uric acid, UA).

Lungs are the organs exposed to a rich oxygen-containing environment, offering a large surface area and blood supply, which makes them susceptible to serious injuries caused by oxidative stress [1,2]. The inflammatory cells (neutrophils and macrophages), fibroblasts, endothelial, and epithelial cells liberate reactive oxygen species (ROS) as the end product of their metabolism [3,4]. In other words, oxidative stress is the result of an imbalance between the oxidants and antioxidants, as given in Figure 1, resulting in the overgeneration of free radicals or ROS or reactive nitrogen species (RNS) [5,6].

The major physiological events, for instance, inflammation, hypoxia, and hyperoxia, end up with oxidative stress which contributes to the rise in lung-related complications namely cystic fibrosis (CF), acute lung injury (ALI), pulmonary hypertension (PH), chronic obstructive pulmonary disease (COPD), bronchopulmonary dysplasia (BPD), and lung cancer (LC) along with compromised pulmonary hemodynamics and gas exchange [7]. Moreover, the sources of free radicals are either endogenous (mitochondria, NADPH, oxidases, xanthine oxidases, and endothelial nitric oxide synthase) or exogenous (UV and IR, air pollution, heavy metals, and cigarette smoking) [8,9,10]. The oxidative stress originating from the ROS above further activates several inflammatory cascades, which can enhance the release of cytokines and inflammatory mediators; consequently, more complex lung injury can occur.

CF is classified as an autosomal recessive genetic disorder that results from a mutation of a gene on chromosome VII, which encodes the CF transmembrane conductance regulator (CFTR) [11,12]. In CF patients, the liberation of ROS, combined with the altered functioning of CFTR proteins, leads to an oxidative stress condition within the epithelial cells [13]. As of 4 July 2025, more than 2100 mutations have been listed in the CFTR mutation database [14].

ALI is defined as a hypoxic situation characterized by extensive lung weight gain due to edema and elevated pulmonary vascular permeability [15]. Despite the complex pathogenesis of ALI, the oxidative stress and initiation of inflammatory reactions (release of cytokines) are the key aspects of its development. In recent studies, a 20-fold increase in ROS levels has been reported in the lungs affected by ALI [16]. In ALI, pro-inflammatory cytokines are generally released from M1-like macrophages (a transformation of alveolar macrophages), and this entire event activates monocytes and neutrophils, which further liberate ROS to a great extent, killing pathogens. Consequently, the high production of ROS will elevate normal oxidative stress levels [17,18].

Among the ICU patients who are exposed to different viral infections, especially SARS-CoV-2, the immune reactions will trigger with a greater threshold, and a high mortality rate can be anticipated in this particular case [19]. Oxidative stress aberrantly activates the innate immune response and inflammatory cytokines, which are the major contributing factors responsible for multiple deaths [20]. Owing to exposure to COVID-19, the induced oxidative stress can collapse the pulmonary alveoli (pneumonia), followed by multiple organ dysfunction. Concerning this, the uptake of exogenous antioxidant therapy can neutralize the generated ROS and strengthen the immune system during COVID-19 [21,22].

PH is another complication, especially in the case of newborns, characterized by constriction and increased pressure in the pulmonary arteries, which undergo remodeling [23]. In this particular disease, the pulmonary vasoconstriction takes place due to an excessive release of ROS and RNS, which can inactivate various enzymes and vasodilators involved in nitric oxide pathways, consequently leading to persistent PH [24]. In this scenario, the elevated blood pressure exerts a burden on the right side of the heart, followed by right heart dysfunction, which puts the life of the patients in danger [25]. It is worth noting that there is no specific treatment for PH, and patients can temporarily rely on vasodilator agents. In addition, a median survival of three years after diagnosis was recorded in patients with PH, and, finally, lung transplantation was considered the definitive treatment, again with severe clinical complications [23,26].

Among PH, pulmonary arterial hypertension (PAH) has to be mentioned. Precisely, PAH is classified as WHO Group 1 PH, representing a distinct subtype within the broader category of PH. Group 1 PAH is characterized by a specific set of pathological changes in the pulmonary arteries, leading to increased pulmonary vascular resistance and elevated pulmonary artery pressure. PAH can be idiopathic (unknown cause), but also be inherited or associated with other conditions, including sickle cell anemia, connective tissue diseases, portal hypertension of the liver, HIV infection, and congenital heart disease. PAH can even occur after exposure to drugs and toxins and is occasionally related to pulmonary veno-occlusive disease or persistent PH of the newborn [27,28]. Furthermore, PH is associated with elevated oxidative stress, so it is important to emphasize that DNA damage and pulmonary vascular remodeling are significant concerns in the lungs of PAH patients. ROS and DNA are considered biomarkers of PAH susceptibility in various subgroups of PAH patients. Currently, there is no effective treatment for PAH [29].

LC occurs when the lungs are exposed to harmful carcinogens, as evidenced by genetic and epigenetic alterations [30]. The malignancies related to the lungs have also been a leading cause of the increase in death numbers in recent years. Squamous cell lung carcinoma and adenocarcinoma LC are more prominent, which are 30% and 40%, respectively, among non-small cell LC subtypes [31,32].

These elevated stress conditions within the cancerous tissues act as a strong driving factor responsible for shifting the free radical balance towards oxidative conditions, which further results in a reduced antioxidant threshold compared to non-cancerous or healthy tissues [33,34]. In this regard, an increase in the level of reactive aldehydes, oxidative modifications in DNA, and nuclear factor erythroid-2-related factor (NRF2) activity, along with a decline in fatty acid levels, has been monitored. Taking this into account, this study has emphasized the relationship between various lipid mediators and antioxidants in different types of LC [32].

BPD is a chronic lung disease primarily affecting premature infants and is a common cause of morbidity and mortality. It is characterized by lung damage and disrupted lung development, often stemming from the need for oxygen therapy and mechanical ventilation in babies with underdeveloped lungs. Therefore, BPD is a chronic disease persisting beyond hospital discharge and into adulthood. While BPD is not present at birth, it can develop as a complication of other breathing problems, including respiratory distress syndrome (RDS), asthma, and emphysema. Furthermore, BPD is also involved in neurodevelopment, significantly affecting and delaying fine and gross motor skills and language [35,36,37]. In addition, infants with BPD are at high risk of cardiopulmonary sequelae, such as PH and systemic hypertension. The oxidative stress activates the inflammatory cells, especially granulocytes, which potentiates the inflammatory reactions. In this regard, the employment of antioxidants in preterm newborns predominantly exposed to oxidative stress and at risk for BPD represents a logical strategy to ameliorate injury caused by the presence of free radicals [37,38,39].

**Figure 2:**
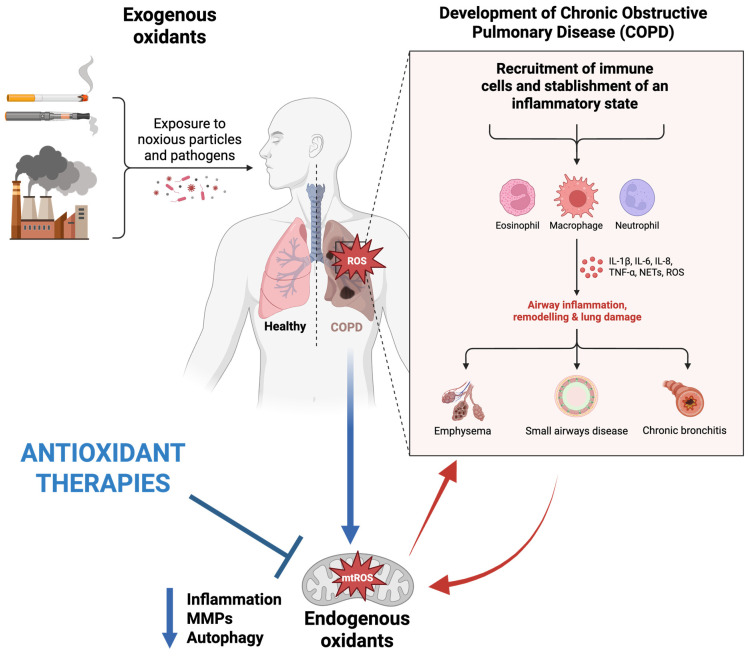
Schematic representation of factors influencing the pathogenesis of COPD and therapeutic effects of mitochondria-targeted antioxidants. Original figure created with www.BioRender.com (accessed on 30 June 2025).

COPD is the third leading cause of death after stroke and cardiovascular disease, characterized by irreversible obstruction in the airflow [40]. Oxidative stress remains a leading concern in COPD, which affects various lung events, including the inactivation of anti-proteases, injury to alveolar epithelial architecture, remodeling, and ultimately, apoptosis. In addition, due to an upsurge in the levels of both oxidants and oxidative stress-related markers, patients experience chronic inflammation, fibrosis, and blood in the airways, which ultimately damages the lung parenchyma [10,41,42]. Researchers have reported that in COPD patients, an elevation in oxidative stress conditions and a decline in glutathione concentrations within the bronchoalveolar lavage fluid are present [43]. Considering that air pollutants represent one of the primary sources of oxidative stress, they are known to be involved in the development of many respiratory conditions, including COPD. The role of pollutants in the pathogenesis of COPD has been presented in Figure 2.

It is a well-established fact that the intracellular level of ROS is stabilized by several enzyme or non-enzyme-linked cellular processes. The primary enzymes involved in the breakdown of oxidants are ascorbate peroxidase, glutathione peroxidase, metallothionein-3, ferritin heavy chain, catalase, and superoxide dismutase. Non-enzymes that destroy ROS by interrupting free radical chain formation through the building of a metal-protein complex [44,45].

The mitochondria are primarily engaged in the development of oxidative stress in all previously highlighted lung-related complications. When examining the cellular impact, ROS exert a more profound detrimental effect on mitochondrial DNA, the genetic material encoding mitochondrial proteins, than on nuclear DNA. The adverse effects observed include not only DNA damage but also impaired mitochondrial electron transport chain activity and altered membrane potential [46,47]. In addition, the hydroxyl ion is highly reactive towards lipids among the other ROS members, responsible for lipid peroxidation and protein fragmentation [48,49]. Moreover, this event can also influence the inflammatory and apoptotic pathways, with increased permeability of the outer mitochondrial membrane [50,51].

## 2. Role of Antioxidants in the Management of Lung-Related Complications

Taken together, both oxidative stress and impaired antioxidant mechanisms are key elements in the development of injury, which can permanently damage the lungs [18]. To minimize this ROS-induced lung destruction, the exogenous intake of antioxidants could potentially retard the deterioration of lung functioning [52].

Antioxidants are the moieties that exhibit remarkable radical scavenging potential, neutralizing ROS by donating an H-atom to free radicals. They can be employed as an effective therapy to address oxidative stress-induced lung complications [52,53]. As mentioned earlier, oxidative damage to lipids, proteins, and nucleic acids can be prevented by neutralizing free radical species with antioxidant substances [5,54,55].

The role of antioxidants in counteracting oxidative stress-related pathologies is summarized in Table 1.

Building on this, polyphenols are naturally derived, plant-based secondary bioactive compounds found abundantly in fruits, vegetables, grains, and herbs, known for their potent antioxidant and anti-inflammatory properties that offer therapeutic benefits in various lung diseases [65]. They help reduce oxidative stress and inflammation, which are key factors in lung conditions such as ALI, COPD, asthma, pulmonary fibrosis, PH, and LC [66]. These compounds work by neutralizing harmful free radicals, suppressing pro-inflammatory cytokines like TNF-α, IL-1, and IL-6, and blocking critical inflammatory signaling pathways such as NF-κB and mitogen-activated protein kinase (MAPK) [66]. For instance, flavonoids, such as kaempferol or pinocembrin, alleviate lung inflammation by inhibiting the phosphorylation of signaling molecules (ERK1/2, JNK, p38 MAPK) and reducing inflammatory cell infiltration in lung tissues [67].

In COPD patients, dietary polyphenols improve lung function parameters (e.g., FEV1/FVC ratio) and reduce oxidative damage by boosting antioxidant enzymes such as superoxide dismutase and glutathione, modulating gut microbiota, and activating pathways like 5′ adenosine monophosphate-activated protein kinase (AMPK) to prevent airway remodeling. Epidemiological and clinical studies endorse the lung-protective effects of diets rich in polyphenols. Meta-analyses reveal that higher consumption of fruit-derived polyphenols, including anthocyanins, correlates with enhanced pulmonary function (e.g., FVC, FEV1) and decreased inflammatory markers in both healthy individuals and patients with lung diseases [68,69]. Additionally, polyphenols help regulate immune cell recruitment and mitigate chronic lung inflammation, indicating their promise as complementary treatments for respiratory disorders driven by oxidative stress and inflammation [65]. Furthermore, polyphenols exhibit chemopreventive potential in LC by regulating phase I and II detoxifying enzymes, preventing DNA oxidative damage, and modulating cell survival pathways. Epidemiological research supports an inverse relationship between high polyphenol consumption and LC risk in at-risk populations, indicating their role in reducing carcinogenesis through antioxidant and antimutagenic mechanisms [30].

In particular, polyphenols were shown to exert beneficial effects against LC by regulating the expression of microRNAs via different mechanisms, especially by scavenging ROS production [64].

Studies show that several polyphenols, such as epigallocatechin gallate (EGCG), quercetin, resveratrol, and curcumin, can reduce the release of ROS involved in the dysregulation of miRNA regulatory pathways crucial for cell survival, proliferation, and apoptosis [70,71]. Notably, these polyphenols modulate the expression and activity of molecules within ROS-mediated anticancer pathways. This is achieved by increasing the expression of tumor-suppressive miRNAs while simultaneously decreasing the expression of oncogenic miRNAs [72]. For example, quercetin has been demonstrated to upregulate miR-16, thereby contributing to the suppression of anti-apoptotic proteins such as Bcl-2. For instance, curcumin has been observed to elevate the levels of several tumor-suppressive miRNAs, including miR-34a and miR-101, concurrently downregulating the oncogenic miR-21. Furthermore, epigallocatechin gallate (EGCG) has been shown to increase miR-210, leading to the inhibition of LC cell proliferation [72,73]. Moreover, by modulating miRNAs, polyphenols can block pro-tumorigenic redox signaling pathways, such as PI3K/Akt, MAPK, and apoptosis-related cascades.

The polyphenols, namely quercetin and curcumin, exhibit numerous alterations in cellular processes after binding to microRNAs, which interrupts the invasion and migration of lung adenocarcinoma cells via the upregulation of microRNA-16 [70]. Other examples include the polyphenols Apigenin, thanks to its ability to induce apoptosis via the upregulation of microRNA-34a-5 [74], hesperidin, which inhibits cell proliferation by targeting microRNA-132 [75], and ailanthone, which induces autophagy by upregulating microRNA-195 [76]. Besides their ability to regulate miRNAs via an antioxidant mechanism, polyphenols can suppress cancer-promoting miRNAs or upregulate tumor-suppressor miRNAs via an epigenetic mechanism acting as inhibitors of DNA methyltransferase (DNMT) and histone deacetylase (HDAC) [77].

Firstly, even if the cause and pathogenesis of COPD are not fully understood, cigarette smoke has been reported as the primary etiologic factor in the progression of COPD [78]. The smoke produced from cigarettes can alter various mechanisms involved in inflammation, ultimately generating oxidant substances that can damage vital lung functioning [79]. To neutralize the oxidants, the potential of polyphenols available in apples has been investigated against oxidative stress-based ALI induced via exposure to cigarette smoke. Interestingly, the antioxidants significantly counteract lung injury by reducing oxidative stress and p-P38 expression levels [80].

Secondly, the pathogenesis of vascular aging and COPD is also associated with oxidative stress and is attributable to the deterioration of numerous anti-aging pathophysiological events. These mechanisms can promote the activation of sirtuin, which affects the Klotho protein-fibroblast growth factor (FGF) pathway as well as the induction of certain epigenetic modifications that collectively result in mitochondrial dysfunction [81].

Similarly, another subtype of RNA, known as non-coding RNA, also plays a significant role in regulating hypertension by expressing specific genes, such as through splicing, RNA editing, translational inhibition, and mRNA degradation. This subtype of RNA is responsible for controlling chromosome dynamics and is engaged in the expression of various diseases, especially hypertension. Interestingly, it has been found that polyphenols can target non-coding RNA and regulate its expression in hypertension [82].

## 3. Role of Nano-Scale Drug Delivery to Treat Lung Diseases

Interestingly, the therapeutic effects of natural and synthetic antioxidants have been demonstrated in animal model-based studies. Nonetheless, their reproducibility at the clinical level remains a considerable concern, which may be attributed to their limited absorption, concentration, and half-life [83].

The route of administration considerably impacts the efficacy and safety of the drug delivery. The parenteral route of administration remains a significant option for delivering biopharmaceuticals to the lungs for targeted delivery. Nonetheless, enduring therapy is often unable to maintain clinical suitability and patient adherence [84]. Apart from this, drug targeting has numerous obstacles associated with poor bioavailability, low aqueous solubility, and degradation due to the hepatic first-pass effect [85].

Pulmonary delivery involves the direct transportation of actives to the lungs, where the drugs either accumulate or are used as a medium to treat local and systemic diseases. The lungs present a large surface area of thin epithelial membranes, which are smooth and facilitate the exchange of drug molecules between the air and blood, thereby allowing therapeutic moieties to reach the target site at the appropriate concentration without any deterrents [86,87]. Therefore, the airway route is an attractive approach for drug transportation, reflected in its non-invasive nature, which not only helps in managing damaged lung tissue but also enhances the systemic absorption of specific drug molecules directly from the lungs [88]. In addition, the first-pass metabolism, plasma binding, and systemic distribution can be avoided by adopting pulmonary-based drug delivery as a route of intake [89].

In addition, the instantaneous onset of action, along with higher efficacy, can be expected by avoiding systemic side effects at minimal doses compared to other routes of administration [90]. The drug molecules entering through the pulmonary route exhibit short-term action due to their immediate clearance; hence, prompt absorption is possible [91].

Concerning the translocation of drug moieties, it occurs via transcytosis into the epithelial cells and across the respiratory epithelia into the interstitium and then finally reaches to blood and lymph. It is worth highlighting that drugs reach the blood faster than other non-invasive modes of administration. In addition, short pulmonary exposure to inhaled medicines [92,93]. Furthermore, the small lipophilic drug molecules from the air lodge in the lining fluid of the lungs due to their significant miscibility with the fluid in the lung lining and are countered by lung surfactants first, which further facilitate the dissolution process, resulting in enhanced absorption. However, in the case of larger molecules, the absorption might be slower, but higher bioavailability can be anticipated from pulmonary delivery comparatively to non-invasive routes [94].

In the case of inhalation LC therapy, the biodistribution pattern of active molecules at the desired site can be modified to achieve higher accumulation. In this regard, the inhaled particles undergo lymphatic circulation and create a depot in the lymph nodes. It will allow the drug molecules to redistribute in the peripheral respiratory tract, allowing not only the uptake of poorly soluble drugs but also counteracting the cancerous cells that have metastasized to other neighboring organs [95,96]. Additionally, due to targeted delivery, the dose of anticancer agents can be minimized. At the same time, in the systemic application, high doses can toxicate the non-targeted organs as well [97].

Despite the numerous advantages, the lungs also present challenges, as the airway is fully equipped with defense mechanisms to prevent the entrance of exogenous particles into the respiratory tract, which limits pulmonary drug uptake. Therefore, the air-passage and clearance pathways are highly crucial elements that determine the availability of the drug molecules at the site of action [95,98]. For instance, inhaled particles are cleaned away in two ways. Firstly, cilia are present in the trachea and bronchi from the lungs along with mucus, and secondly, via phagocytosis of alveolar macrophages. In this manner, macrophages can adversely modify the fate of drug-carrying inhaled particles by triggering the immune response cascade. In other words, antigens taken up by macrophages are generally presented to the T lymphocytes, and co-factors in the lymph nodes may further activate or suppress the events related to humoral immunity [94,99].

**Figure 3:**
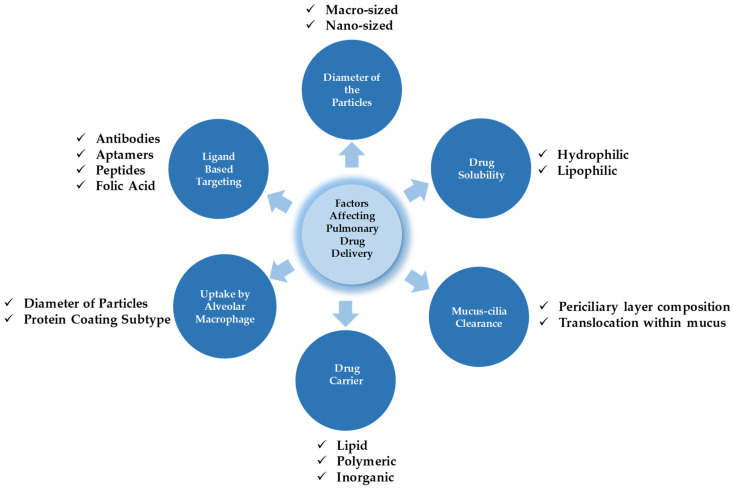
The interplay of factors dictating pulmonary drug targeting, retention, and biotransformation.

Therefore, drug particles for pulmonary administration should be capable of overcoming these physiological barriers and reaching the desired site without losing their integrity [100]. Some of the crucial parameters affecting drug deposition and its metabolism are presented in Figure 3. The major factors affecting drug deposition and metabolism generally depend on the diameter of particles, the solubility of the drugs, mucus-cilia clearance, the type of drug delivery system (DDS), the tendency of drug transporters toward alveolar macrophage uptake, and finally, cell specificity with the aid of a ligand approach.

Moreover, another important barrier that needs to be overcome in the lungs is the pulmonary surfactant, a naturally occurring phospholipid–protein mixture that coats the alveolar surfaces in the lungs and plays a critical role in modulating the adsorption and fate of inhaled nanocarriers. Indeed, this surfactant is enriched with components such as dipalmitoylphosphatidylcholine (DPPC) and surfactant proteins (SP-A, SP-B), which can rapidly adsorb onto the nanoparticles’ surface when they reach the alveolar space. As a consequence, a “surfactant corona” may be formed on the surface of nanoparticles, which changes the surface charge, hydrophobicity, and steric properties of the systems, thus affecting nanoparticle dispersion and aggregation [101,102].

Aggregation of nanoparticles can reduce their deposition and uptake by lung cells, and the interactions with surfactant may impact how deep the nanoparticles can travel within the lung [103,104].

To overcome the pulmonary surfactant barrier, pulmonary surfactant can be strategically utilized to improve drug delivery across the air–liquid interface of the lungs. Indeed, surfactant-mimicking modifications help particles remain at the air–liquid interface, enhancing local drug concentrations, and formulations that combine drugs with surfactant have been shown to enhance drug uptake into lung cells and reduce inflammation in models of lung injury [104,105].

Taken together, tough physiological barriers, lack of cell specificity, and the inability to reach the optimum drug concentration at the site are critical concerns associated with the conventional mode of lung therapy, which can be addressed and avoided by adopting nanotechnological approaches in the area of drug delivery [106].

Nano-sized vehicles can deliver the therapeutic cargos to the lungs to cure both local and systemic diseases. These nano-sized carrier systems are fabricated from organic, inorganic, and/or hybrid materials and are not only capable of delivering natural/synthetic drug molecules but also can convey complex macromolecules, including DNA, proteins, and peptides, by targeting the desired site of action [107,108]. More precisely, nanostructure-enclosing therapeutic agents can achieve sustained release, active or passive drug targeting, uniform dose distribution, and multiple doses can be embedded in a single unit. The controllable diameter, modifiable surface charge, and properties make them very attractive candidates [107,109,110].

The success of nanotechnological products in terms of commercial viability, with promising results, is clearly evident from the already launched Abraxane^®^ and Pazenirl^®^. These are FDA-approved nano-formulations employed to treat LC [111].

Particularly, in the case of lung delivery, the aerodynamic diameter of the particles plays a crucial role, as particles larger than 5 µm are likely to settle superficially. In comparison, smaller particles are taken up by macrophages and reach the deeper lung domains. It should be underlined that the rate of clearance is considerably higher than mucus-cilia clearance [112,113]. The nanostructures, once lodged in the lungs, undergo deep dissolution followed by immediate drug release, which directly depends on the low molecular weight and lipid solubility of the drug candidate [95,114]. However, the interaction of the nanomaterial with pulmonary surfactant and the different components of the lining fluids of the upper and lower respiratory tract would result in the formation of a protein corona that can affect the dispersion of the nanoparticle as described above. To overcome this problem, pulmonary surfactant can be used to enhance drug delivery across the air–liquid interface of the lungs [104,105].

Fascinatingly, the solubility profile of water-soluble drug molecules can also be amended by encapsulating them into respective nano-vectors. Therefore, apart from surface tuning, diameter reduction, and biocompatibility, nano-encapsulation of hydrophilic therapeutic moieties can also be achieved with the aid of nano-carriers [106,115].

Therefore, nano-sized particles can be an alternative approach to attain an effective pulmonary-based drug delivery. Moving further, apart from the diameter, charge properties on the surface of particles cannot be overlooked because the specific surface charge allows them to permeate through the lung tissues to a considerable extent. The negative charge on the cell membrane can be countered by applying a cationic charge to the surface of the drug particles. Nanotechnology offers the possibility of modifying the surface charge by employing suitable cationic surfactants to enhance uptake at the cytoplasmic level. However, their higher concentration may impart toxic effects, which need to be investigated thoroughly [114,116].

Despite the impressive success of nanomedicine in the domain of drug delivery, some targets remain to be accomplished. Specifically, in cancer treatment, the major obstacle still exists in terms of drug loss before reaching the target site. Only 0.7% of the injected dose in the form of nanoparticles reached the tumor cells, while a significant fraction is usually undergoing primary reticuloendothelial system (RES) clearance, primarily in the liver and spleen. Only a limited number of studies focusing on the endothelial-targeted antibody approach for avoiding undesired nanoparticle uptake and clearance have been published [117]. Table 2 summarizes the advantages and challenges of pulmonary delivery of nano-scale systems in the treatment of lung diseases.

Taken together, nano-scale drug delivery can be auspicious and can overcome all the shortcomings related to conveying therapeutic active moieties by conventional methods. Moreover, nanotechnology offers numerous possibilities for treating lung diseases with minimal dose and toxicity.

## 4. Role of β-CD in Pulmonary Drug Delivery of Antioxidants

The donut-shaped cyclodextrins (CDs) are a series of macrocyclic units containing oligosaccharide units α-1,4-linked D (+)-glucopyranose, which act as molecular containers explored for biomedical applications by researchers globally. The CDs are mainly of three types, namely α-, β-, and γ-CDs, in which α-1,4 glycosidic bonds conjugate 6, 7, and 8 D-glucose units [118].

The water solubility of β-CDs is lower than that of all CDs, which is responsible for the strong internal Hydrogen Bonding in its crystal state. The chemical modifications are carried out on β-CDs to enhance water solubility, and examples of these modified products include hydroxypropyl-β-CDs, sulfobutylether-β-CDs, and epichlorohydrin-β-CDs. In addition, the melting points of all the aforementioned CD subtypes lie between 240 °C and 265 °C [119,120].

Interestingly, CDs are composed of both a hydrophilic surface and a hydrophobic central cavity, with the capability of enclosing poorly soluble drugs; hence, the solubility, stability, and bioavailability of the drugs can be improved upon loading onto CDs [15,121,122].

The chemistry of the CDs makes them very attractive as a drug transporter, as they possess two different types of hydroxyl groups (primary at position 6, while secondary at positions 2 and 3) located at the peripheral edge, which is responsible for providing a hydrophilic exterior. On the contrary, oxygen atoms in the glycosidic hemiacetals and C-H units comprise a hydrophobic core [123,124].

**Figure 4:**
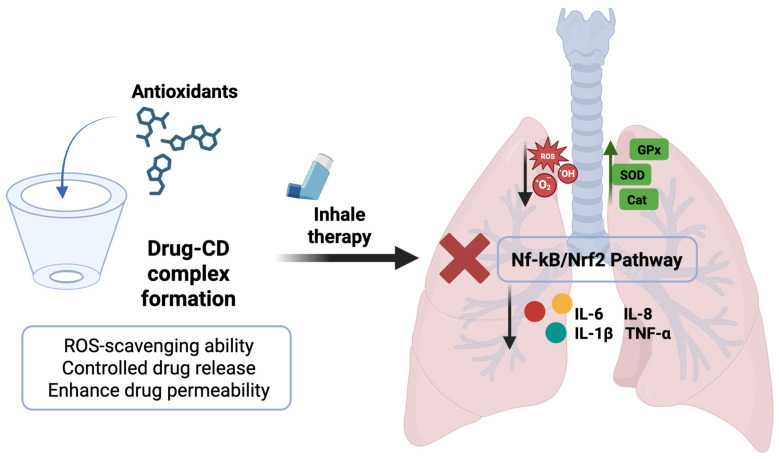
Scheme illustrating the formation of an antioxidant’s inclusion complex and cyclodextrin frameworks with uniform inhalable particle size for the treatment of ALI. Original figure created with www.BioRender.com (accessed on 2 July 2025).

The possibility of adopting a CD-based drug delivery strategy in the lungs, as well as its involvement in the antioxidant mechanism and the inflammatory cascade, is illustrated in Figure 4. Moving ahead, the researchers have also explored the possibility of combining two different types of CDs, namely, α-CDs and 2-hydroxypropylβ-CDs (HP-β-CDs), to achieve a synergistic effect with enhanced solubility of cyclosporine. Similarly, the same trend was observed in the case of dexamethasone, where the additive impact of γ-CDs and HP-γ-CDs has been examined [118].

CDs are edible and usually safe for human consumption and are used commercially at a massive scale in pharmaceutical formulations. More precisely, hydroxypropyl-β-CDs (HP-β-CD) is an FDA-approved ingredient that possesses high solubility (>500 mg/mL) in aqueous medium and low toxicity toward biological membranes. HP-β-CDs and sulfobutylether-β-cyclodextrin are the most widely explored and researched CD-based drug delivery agents [125,126].

Numerous studies have been published in recent years, reflecting the promising potential of β-CDs in carrying antioxidants. Among all, there is research carried out on the β-CDs complex to treat ALI induced due to massive exposure of lungs to bombs and explosive material during army and firefighter operations. In an elaborative manner, bomb smoke is enriched with ZnCl_2,_ which affects the lungs badly on inhalation at high doses, giving rise to ALI followed by acute respiratory distress syndrome [127].

Resveratrol is a polyphenol explored for its role in managing oxidative stress, inflammation, and apoptosis, which is developed in response to various lung injuries; however, its poor solubility, stability, and bioavailability pose significant obstacles [56,128]. The encapsulation of resveratrol in β-CDs complex successfully improved the bio-profile of polyphenols in terms of healing pulmonary edema by maintaining blood vessel integrity and restoring respiratory function within a short time [129].

Pulmonary delivery of CDs results in rapid systemic absorption, broad extracellular distribution, and efficient renal clearance, minimizing tissue accumulation, including in the lungs. Animal studies report high bioavailability (up to 80% after lung instillation), confirming systemic circulation entry via pulmonary routes. CDs primarily distribute in extracellular fluids and are rapidly renally excreted [130]. Preclinical studies show minimal pulmonary toxicity of β-CD at therapeutic doses; for example, Yokohira et al. found only mild lung toxicity with no acute damage or fibrosis after intratracheal instillation in rats (2 mg/rat), supporting safety as a lung delivery excipient [131]. Nebulized native and modified CDs were found not to cause acute lung toxicity at relevant drug-delivery concentrations, though mild lymphocyte increases in bronchoalveolar lavage fluid (BALF), suggesting low-grade immune activation without cytotoxicity or fibrosis. Moreover, while short-term inhalation of β-CDs or hydroxypropyl/γ-CD derivatives did not cause macrophage accumulation or histological lesions in murine models, higher doses or β-CD derivatives like methylated β-CD were found to increase cytotoxicity in vitro [131,132].

Pharmacokinetic studies in rabbits following intratracheal and intravenous administration found β-CD and derivatives with high systemic bioavailability (66–80%) after lung delivery, rapid absorption, renal elimination, and minimal tissue retention. For instance, HP-β-CD, despite its lower absorption from lungs, was efficiently transferred to systemic circulation and clearance with negligible organ retention [133]. Other studies confirm low tissue accumulation after parenteral (including pulmonary) delivery, with a renal excretion > 90% and near-complete elimination within 24 h [134]. Novel derivatives like thiolated HP-β-CD enhance local drug solubility, retention, and absorption without increasing systemic persistence despite mucoadhesive properties, as carriers remain rapidly cleared by absorption and renal excretion [135,136].

Beyond drug delivery, β-CD and modified CDs exhibit direct therapeutic effects in lung diseases, including anti-inflammatory and antioxidant properties, improved surfactant function, antiviral barrier, and anti-fibrotic activities, thus contributing to lung tissue stabilization [136]. For instance, modified β-CDs, notably HP-β-CD-SH, can form nanoaggregates that stabilize lung epithelium and reduce oxidative stress/inflammatory markers, whereas HP-β-CD formulations were found to reduce viral attachment and replication, serving as respiratory barriers. Recent 2024 studies, including SARS-CoV-2 models, show HP-β-CD lowers viral load and lung inflammation by disrupting viral entry, demonstrating direct antiviral and immunomodulatory effects [137,138].

Inhaled β-CD can reduce inflammation and fibrosis by regulating profibrotic proteins and collagen in pulmonary fibrosis models. Its anti-fibrotic effect is enhanced when combined with compounds like tetrandrine (as inclusion complexes), reducing hydroxyproline and improving survival in animals [139].

Studies of pulmonary surfactant dysfunction in CF, acute respiratory distress syndrome (ARDS), and bronchiolitis reveal impairment due to cholesterol and oxidized phospholipids. Methyl-β-CD restores surfactant function and exerts anti-inflammatory effects, supporting inhaled CDs’ therapeutic potential for improving lung function and reducing inflammation across pulmonary diseases, enabling rapid deployment for acute injuries [140,141].

Another interesting approach for lung delivery of drugs is represented by CDs-based nanosponges (CD-NS). Recently, CDs-NS have been explored as a promising DDS for pulmonary administration, potentially improving the effectiveness and targeted delivery of drugs to the lungs. These macrostructures are crosslinked CDs characterized by a three-dimensional nanostructured network [142,143,144].

Notably, these complex macromolecular structures are composed of individual CD and nanochannels created between cross-linked CD units and cross-linkers. Therefore, CD-NS are colloidal carriers highly porous and able to maintain the properties of CDs, such as bioavailability, non-toxicity, biodegradability, and controlled release [145,146,147]. CD-NSs retain the ability to encapsulate poorly water-soluble drugs within their internal nanocavities, but also the possibility of achieving drug loading by forming non-inclusion complexes thanks to the presence of tiny mesh-like structures obtained through cross-linking [148].

As an example, the study of Abou Taleb and colleagues focused on the development of CD-NS for loading the flavonoid quercitrin to enhance, at the same time, the drug solubility and its activity against LC as well as the SARS-CoV-2 virus. The results obtained confirmed the expectations, allowing the possible use of CD-NS as a basis for further animal studies [142].

### 4.1. β-CDs-Drug Conjugate Grafted Surface Modification

The performance of a CDs-based carrier system can be altered chemically, supporting better control over the drug release mechanism, mechanical characteristics, and response threshold to stimuli. The most significant result of these modifications is Cyclosert^TM^, a linear CDs-DDS designed and launched commercially, which has been conjugated to the alkaloid camptothecin for anticancer therapy [122,149].

However, the toxicity associated with the design of β-CDs is a significant concern, which is one of the obstacles to their clinical transition. Concerning this, hydrophilic β-CDs, a toxicity-free derivative form of existing β-CDs, have been synthesized, wherein conjugation of hydroxypropyl groups of β-CDs has disrupted the intermolecular H-bonding in secondary hydroxyl groups of non-modified CDs, making possible the formation of H-bonds with the surrounding water molecules. This H-bonding has not only improved the aqueous solubility of the conjugate mentioned above but also minimized its toxicity [121].

It has been found that in many diseases, the misfolding of various proteins, along with impaired amyloid formation, can exhibit detrimental effects on different organs and tissues. The β-form CDs carrying β-amyloid have significantly reduced the aggregation of multiple proteins, including amyloid-β, insulin, recombinant human growth hormone, prion protein, transthyretin, and α-synuclein, as well as some multimeric enzymes, at very low concentrations in both in vitro and in vivo experiments. It is worth noting that α-, β-, and γ-CDs exhibit different anti-aggregant activities in various aromatic moieties [150,151]. Taking this further into account, different scaffolds containing hydroxyquinoline-cyclodextrin conjugates have been developed to establish synergism, supporting two mechanisms simultaneously: an antioxidant effect and a protective effect against amyloid-β-aggregation [151].

**Figure 5:**
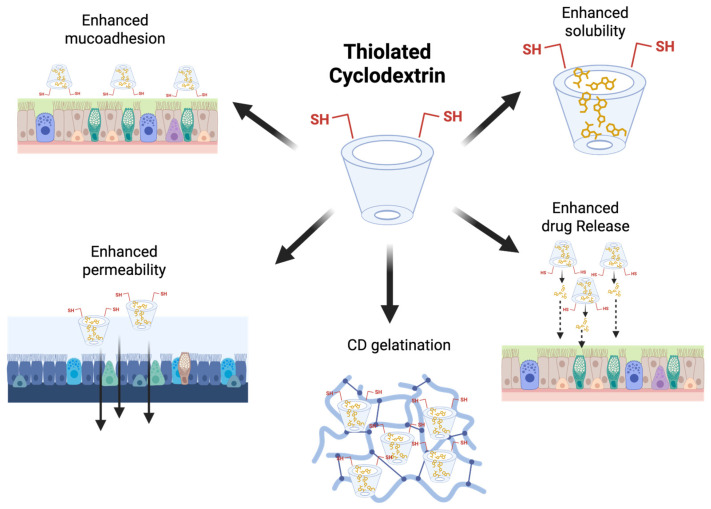
Schematic summary of the main properties of thiolated CDs. Original figure created with www.BioRender.com (accessed on 30 June 2025).

The mucus present in the nasal and lung cavities is composed of water, salts, and various glycoproteins, which protect the epithelium lining from external mechanical and enzymatic damage. It offers a formidable barrier to drug penetration [152]. To achieve improved mucoadhesive properties, thiolated HP-β-CDs have been fabricated, which can form disulfide bonds with cysteine-rich subdomains of a well-known glycoprotein; thus, pulmonary absorption can be enhanced. This strong affinity toward mucus will prolong the residence time of the drug moieties [123,152]. In the continuation, another complex named thiolated 2-methyl-β-CDs was employed in the fabrication of nanoaggregates enclosing dexamethasone. This complex was first synthesized via a microwave-assisted process, a straightforward method shown in Figure 5 [151].

Biofilms are the dispersion of immobile microbial colonies that produce polysaccharides, proteins, and nucleic acids based on a water-soluble matrix, which offers hindrances to the effectiveness of antibiotics due to poor permeability [153]. In CF, the risk of bacterial infection is significantly higher, which can subsequently lead to biofilm formation. This event is further taken up by the emission of quorum-sensing signals, which indicate the presence of bacterial strains. These different strains colonize to form biofilms, which a sputum test can confirm [154]. CDs can interrupt this Quorum Sensing inhibition (QSi) mechanism. To resolve this issue, long-chain alkylthio CD derivatives have shown an intensive inhibitory effect on the quorum-sensing system of *A. fischeri*. Hence, this modified CD system can interfere with QS signaling and control bacterial infections in CF [155].

Sulfobutyl ether-β-CDs (SBE-β-CDs) can enhance the aqueous solubility of lipophilic actives and are found to be safer compared to other derivative products of CDs. This complex was further conjugated with bedaquiline (FDA-approved anti-mycobacterial moiety) and explored further for the treatment of non-small cell LC. This approach is a very effective inhalation therapy tool for inducing cytotoxicity in tumor cells [156].

Following the COVID-19 pandemic, numerous targets have been achieved to combat respiratory viral infections, and it has been demonstrated through in situ RNA mapping that nasal susceptibility to viral infections is significantly higher than that of the lungs. The CD-based formulations allow us to block the attachment of viral moieties to the nasal lining. Taking this into account, two CDs, HP-β-CD and HP-γ-CD, were then investigated for their antiviral effects using SARS-CoV-2 pseudotypes. The significant reduction in viral load and inflammatory signaling in both Calu-3 cells and the K18-hACE2 murine model has been monitored [137].

Delamanid, an anti-tuberculosis medication, faces challenges such as poor solubility. Interestingly, solubility enhancement was 54-fold with HP-β-CDs, while 27-fold and 13-fold enhancements were achieved with SBE-β-CDs and HP-ɣ-CDs, respectively. In addition, the complex has shown significantly higher anti-bacterial activity against M. tuberculosis than a non-encapsulated drug solution alone [157].

Apart from drug delivery, chemically modified β-CDs are also helpful in radio-imaging for the early detection of cancer. A study emphasizing the diagnostic ability of CDs was conducted, in which randomly methylated β-CDs were conjugated with prostaglandin E2. It is a well-established fact that prostaglandin E2 is typically overexpressed in cancerous cells. The molecular dynamics-based docking tool has also confirmed the chemical affinity between β-CDs and various enzymes and proteins within the physiological environment [118,158].

By considering all the merits of CDs, it can be noted that the encapsulation of natural molecules in CDs could be an impressive approach, as most natural extracts face solubility issues and are prone to degradation due to non-enzymatic and enzymatic dioxygen oxidation. Therefore, all the capabilities of CDs can be leveraged to overcome these challenges [159]. Recent developments in CDs and their derivative-based antioxidant encapsulation are summarized in Table 3.

### 4.2. β-CDs-Based Hybrid Framework

It is worth mentioning that CDs, as a system, possess numerous qualities and yield auspicious results in physicochemical, in vitro, and in vivo evaluations. Fascinatingly, CDs also have great potential as a secondary carrier. This section of the review will explore the possibility of conjugating the primary vehicle (lipid, polymer, or inorganic-based nanosystem) with a CD’s inclusion cage, where the combination of the two systems can lead to improved performance. Surprisingly, the tertiary system can also be introduced as a gel in the management of the wound-healing process [166].

Metal–organic frameworks technology is a combination of metal ions and organic ligands, seen as a promising tool for modulating the shape, dimensions, aggregation, and composition of particles. Wherein, multiple ligands can be conjugated with metal ions; interestingly, this conjugate can be further incorporated into the supermolecular β-CDs complex, which will hold both properties in one system. The combining effect will not only allow for higher drug loading with enhanced stability and bioavailability but also provide the possibility to attach ligand markers for cell-specific targeting [167,168].

The metal ions are biocompatible, and no toxicity concerns have been reported so far. The coordination of CDs with metal ions such as calcium, potassium, titanium, silver, iron, and yttrium has been reported [169]. It is worth mentioning here that among all the discussed CDs, the presence of –OCCO- in γ-CD makes it more suitable for interaction with metallic structures; it is also non-toxic and is considered as one of the first porous crystals that comprises the amphiphilic nanopores [170,171]. Furthermore, the employability of cetyl trimethyl ammonium bromide and polyethylene glycol 20000 is not only helpful in regulating the shape and size of the particles but also enhances the reproducibility of the synthesis process [172,173].

Topotecan, an anticancer drug, is highly effective against LC encapsulated in a metal–organic framework complex. Efficient loading, along with sustained release, was achieved at the local site, specifically in the lungs, after intravenous administration. The remarkable resistance to hydrolysis was monitored, which was significantly increased by many folds, thereby substantially extending the half-life of the drug. The complex has demonstrated noteworthy anticancer activity by inhibiting the migration and invasion of B16F10 cells [174].

The porogens, such as ammonium bicarbonate, poly (vinyl pyrrolidone), and Pluronic F127, are the agents utilized in the production of porous materials, which present inevitable obstacles to achieving uniformity in pores and batch-to-batch reproducibility. Moreover, the porogens need to be evaporated, which requires high-energy heat treatments that can further harm the thermolabile encapsulated therapeutics [175,176]. Yixian Zhou and team emphasized that the use of porogens can be avoided by considering CDs-based metal–organic frameworks (CD-MOFs) carrying ketoprofen prepared by the vapor diffusion method in the management of pulmonary inflammation. The proposed complex exhibits improved aerosol properties, a higher drug loading capacity, and enhanced lung deposition, as confirmed by the rat model [177].

Pulmonary function test was conducted on rats to evaluate the effects of intratracheal administration of CD-MOF, drug-loaded CD-MOF (CD-MOF-K), and a commercial formulation. A lung function system, coupled with a ventilator and plethysmograph, was used to assess lung resistance (RL) and dynamic lung compliance (Cdyn) at one and eight hours post-administration. The results showed no statistically significant differences in either RL or Cdyn between any of the treated groups and the untreated (blank) control group at either time point [177].

One step ahead, a biological cell can also be utilized as a drug carrier to solve numerous physiological obstacles. Following this point, the surface of endogenous macrophages has been modified using β-CDs and has been explored as a drug carrier for the transportation of Adamantane-modified quercetin-loaded liposomes. Therefore, this involves the invagination of quercetin-loaded liposomes into CD-based modified macrophages that successfully activated the NRF2 pathway to inhibit plaque inflammation [178,179].

A recent study has been published, emphasizing the pulmonary delivery of Cyclosporine A based on γ-CDs metal–organic frameworks. The analysis exploits the vapor diffusion method and multiple modulators to achieve optimum aerosol properties and in vitro results. The results suggest that cubicle-shaped particles, along with reproducible thermal behavior and crystallinity, have been obtained. More precisely, in the case of polyethylene glycol 10000, the enhanced bioavailability has been recorded. The repeated dose also confirmed that no toxicity-related issues have been noted [180]. Xiaoxiao Hu and team have selected cholesterol and leucine as modulators in the case of pulmonary delivery of budesonide to increase the uniformity and flow properties of powders. The cholesterol has significantly improved flow properties with significantly higher encapsulation and minimal cytotoxicity. Furthermore, rhodamine B, used as a marker in fluorescence microscopy, confirmed the adequate deposition of this complex in the deeper portions of the lungs [132]. However, the careful selection of solvent and surfactant can be helpful in preparing a Dry Powder Inhaler (DPI) with controllable size and shape [172].

Nianxia Sun and co-authors have presented a study aimed at β- and γ-CDs metal–organic framework-based pulmonary delivery of allyl isothiocyanate. High drug loading and stable crystalline morphology have been achieved in this particular case. The diameter obtained ranged from 1 to 5 μm, and γ-CDs were found to be deposited effectively in the lungs (fluorescence signals have been recorded in a mouse model) and released 90% of the drug content within the first five minutes. It reflects good lung tolerance as no sign of toxicity has been observed [181].

**Figure 6:**
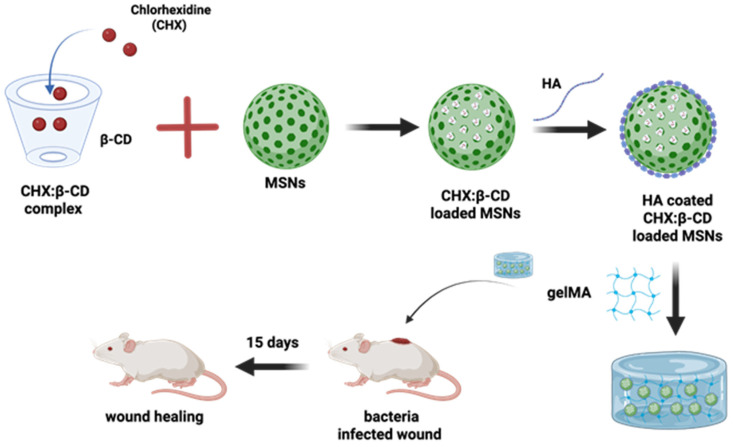
Schematic illustration of the preparation of a hybrid GelMA system combining hyaluronic acid (HA)-coated mesoporous silica nanoparticles (MSNs), which loaded complexes of chlorhexidine (CHX) with β-CDs for the bacteria-infected wound healing. Redrawn figure from reference [166] with BioRender.com.

Going further, the more multifaceted nature of the CD-based complex has been reported by the researchers in a timely manner. This statement can be supported by an example where pH-responsive hyaluronic acid-coated mesoporous silica nanoparticles are further loaded with chlorhexidine/β-CD complexes. This whole system was further put into a hybrid gelatin methacrylate (GelMA) system, which has managed to control the drug release for 12 days with considerable anti-bacterial activity. This gel-based composite has played a potential role in wound healing applications, and its design is shown in Figure 6 [166].

Similarly, the surface modification of gold nanoparticles has been achieved using polyethylene glycol and CDs (α, β, and γ) to encapsulate hydrophobic drugs. In this case, curcumin has been selected as a model drug owing to its poor water solubility. The combined effect of polyethylene glycol and β-CDs has shown the highest encapsulation and a prominent cytotoxic effect against a human LC cell line among all the CDs subtypes [163].

It has been well-explored by researchers that folic acid receptors are overexpressed in the cancer environment. Considering this argument in the treatment of lung and prostate cancer, authors have reported the design of a quantum dots-β-CDs hybrid system wherein a folate ligand has been attached for the cell-specific delivery of the anticancer compound. The complex conjugation was non-toxic and did not affect the performance of the anticancer compound in the encapsulated form. An increased uptake of the carrier has been observed through multiple endocytosis pathways. Hence, the proposed hybrid system possesses self-navigating properties within the physiological tumor environment [182,183].

### 4.3. Concept of Dual Therapy with CDs

Under the influence of serious complications, for example, LC, one regimen of anticancer drugs is sometimes not sufficient to elicit the desired effect. This gives rise to the need to develop a system that allows two drugs to be administered together to enhance their anticancer effect. Interestingly, it is worth stressing here that some of nanosystems (polymeric nanoparticles, micelles, dendrimers, nanocrystals, solid lipid nanoparticles, lipid nanostructured carriers, liposomes, mesoporous silica nanoparticles, etc.) give the possibility to encapsulate two drug moieties without affecting the overall effectiveness However, multi-drug loading onto a carrier still presents some serious flaws as it is very challenging to control drug ratios and their respective release pattern at the site of action owing to different physicochemical characteristics. Additionally, the simultaneous estimation of two drugs for chemical characterization is often a point of debate [184,185].

In this direction, some researchers have attempted to use CDs for combinational therapy. Especially in the case of tuberculosis, ethionamide is used as the second line of treatment; however, it has a low therapeutic index. To enhance the efficacy of this drug, a booster has been synthesized and co-encapsulated in the β-CDs inclusion complex to mitigate the issues of low solubility. The β-CDs managed to enhance the aqueous solubility of ethionamide and booster by 10- and 90-fold, respectively. The loaded drugs did not interfere with each other’s performance [186]. Moving further, in tuberculosis, macrophages host the infection-causing microbes, and a strategic approach to target these macrophages could be beneficial. In this path, two tuberculosis drugs, namely rifampicin and levofloxacin, were complexed with CDs and further linked to curdlan nanoparticles. Curdlan is a glucan acquired from *Agrobacterium*, *Rhizobium*, and *Alcaligenes faecalis*, exhibiting remarkable immunomodulatory and anti-infective properties. With this, a high uptake of drugs has been observed, with major killing of *Mycobacterium smegmatis* residing in macrophages within the four-hour timeframe. Therefore, this type of system can transport hydrophobic drugs into macrophages, providing essential insights into the area of TB-related drug delivery [187,188].

The surge in pulmonary fungal infections cannot be overlooked, as it imparts a significant burden on the healthcare system, with no effective treatment lines available so far. Salomé S. Celi and team workers have performed the co-encapsulation of two known antifungal therapeutic moieties, namely amphotericin B and itraconazole, in the γ-CDs framework. This complex was very effective against aspergillosis and reduced fungal infections in both the lung parenchyma and the upper respiratory tract by avoiding the serious effects associated with Amphotericin B [189].

A CD-based framework functionalized with RGD (a peptide that plays a crucial role in the adhesion of integrins expressed on lung tumor cells) has been proposed for the active targeting of low-molecular-weight heparin and doxorubicin to LC cells following intravenous administration. Therefore, the system will not only exhibit anticancer activity but also aid in the recognition of cancerous cells. The accountable effect has been achieved with five times lower doxorubicin (DOX) concentration; hence, organ-specific toxicity can be avoided with a reduced dose [190,191].

Moving further, working on the co-therapy of two individual drugs, it is also possible to encapsulate the entire extract, which carries multiple active molecules. An attempt has been made to encapsulate resveratrol and polydatin, derived from Polygoni Cuspidati *Radix* extract, for their antioxidant and anti-inflammatory properties. The Polyvinylpyrrolidone/CDs-based electrospun nanofibres managed to enhance the solubility by six-fold compared to the non-encapsulated form, and the activity of the whole extract was preserved [161,192]. Similarly, a blend of gum Arabic, Tween 20, and β-cyclodextrin was used in the embedding of Turkish Oregano Extract, achieving more than 95% encapsulation efficiency for the extract, which comprises rosmarinic acid and carvacrol [162].

The prodrug-based dual therapy has been projected to target colon cancer. 5-Aminosalicylic acid and butyric acid have been loaded into a carboxymethyl-β-CD inclusion complex, which inhibits cell proliferation against SW620 (colon cancer) cells. The long circulation time and high payload at the tumor site have been observed in nude mice bearing SW620 xenografts [193].

Cardiac fibrosis is a condition associated with chronic diabetes. Upon activation, cardiac myofibroblasts begin to accumulate in the extracellular matrix, which is responsible for cardiac dysfunction and ultimately leads to cardiac failure. In this regard, a Chrysin-based supramolecular CDs-calixarene complex has been designed for its anti-fibrotic activity, targeting the selective inhibition of galectin 1, which reduces the profibrotic cascade. In this, the CD complex has significantly enhanced the solubility and bioavailability of both moieties. The combination of both compounds boosts the antifibrotic activity in diabetic-induced rat cardiomyocytes [194,195].

In Section 4.2 and Section 4.3, light has been shed on the possibility of combining two DDS comprising one primary (lipid, polymer, or inorganic-based nanosystem), one secondary (generally, a modified form of CDs) system carrying two different therapeutic cargos. Most of the studies conducted are based on modifications that are performed generally in the secondary system only. Notably, a more sophisticated model has been introduced in which both primary and secondary systems have been modified in a single approach to deliver two different drug molecules efficiently. This can be illustrated further by taking an example, where wheat germ agglutinin-conjugated liposomes with surface-grafted CDs have been utilized for loading two drugs, namely ciprofloxacin and betamethasone, to enhance oral bioadhesion. To mimic periodontal disease modal conditions, oral keratinocytes were infected with *Aggregatibacter actinomycetemcomitans* (*A. actinomycetemcomitans*). Controlled drug delivery for 24 h in saliva, accompanied by a decline in inflammation and remarkable anti-bacterial activity, has been observed [196].

### 4.4. CDs-Based Miscellaneous Therapy Other than Antioxidants in the Pulmonary Complications

#### 4.4.1. Cannabidiols

Cannabidiols are non-psychoactive plant-derived moieties obtained from *Cannabis sativa* that have multiple therapeutic effects, including anticonvulsant, sedative, hypnotic, antipsychotic, anticancer, anti-inflammatory, and neuroprotective properties.

Specifically, during acute cell injury, cannabidiols can decrease the production of inflammatory mediators. It has been suggested by many studies that cannabidiols can downregulate various COVID-19-related receptors and can be adopted as adjuvant therapy in the treatment of COVID-19 [197,198]. Apart from this, Cannabidiols are also helpful in relieving cancer-related pain and have anti-proliferative activity against cancer cells [199].

Besides their numerous merits, poor aqueous solubility is the main obstacle to their clinical applications. For this, a nano-spray solution composed of β-CDs and poloxamer 407 micelles carrying cannabidiol has been designed to reduce the production of cytokines in the inflammation induced by in vitro and ex vivo SARS-CoV-2. It has been concluded that the system was able to stabilize the drug for six months and release the entire loaded drug fraction within five minutes, exhibiting very high permeability across the nasal mucosa. Hence, it can be a promising approach in COVID-related inflammation [195].

#### 4.4.2. siRNA

SiRNA (short interfering RNA containing 21–26 nucleotides) is an effective tool for gene silencing, successfully exploited in pulmonary diseases [200,201]. There is the possibility of introducing β-CDs as a carrier for siRNA, wherein two polycationic lipids, Lipofectamine 2000 and polycationic amphiphilic CDs, are used. The remarkable gene knockdown activity has been obtained from human glioblastoma cells (U87) and prostate cancer cells [202].

It is essential to note that surface charge plays a crucial role in designing the carrier for siRNA. As siRNA is negatively charged, a positively charged delivery system should be selected to ensure better cellular uptake and stability of the cargo. Regarding this, cationic-charged modified CD can be a suitable choice for gene delivery. Molecular dynamics computer simulations, in tandem with isothermal titration calorimetry, can be a beneficial strategy for high stability and minimal toxicity [203]. Furthermore, HP-β-CDs grafted with different grades of polyethylenimine have been selected for the pulmonary administration of insulin, calcitonin, 5(6)-carboxyfluorescein (CF), and fluorescein isothiocyanate dextrans. Considerable pulmonary uptake of these proteins without conferring any toxic effects has been achieved [135]. In continuation, a hybrid system can be proposed using low-molecular-weight polyethylenimine and pH-responsive CDs to load siRNA. This complex exhibits higher cell growth inhibition and apoptosis activation compared to a simple cationic vector [204].

#### 4.4.3. Immunomodulation by Antibodies

It is also crucial to discuss new horizons in the treatment of lung-related diseases, primarily through antibody-based therapies, as they can effectively minimize the burden on the healthcare system [205]. WKS13 is a humanized monoclonal antibody that can counter the spread of SARS-CoV-2. The CDs conjugated to WKS13 have been proposed to target the nasal cavity. It can be effective against a broad range of respiratory viral infections [206]. Anti-human IL-4Rα are the antibodies that can enhance the stability of protein-based moieties, and their biological activity can be a useful inhalation tool in asthma and other respiratory viral infections [207].

## 5. Challenges in Physiological Model Approach-Based Testing

The employability of animals in drug development remains crucial to understand the fate of inhaled actives. In this regard, numerous aspects should be considered, including cost, disease pathology, and immunological similarity to humans. However, the data obtained from pre-clinical studies is not always easy to handle due to dissimilarities in the nasal, tracheobronchial, and deep lung regions across species. Interestingly, rodents are unable to breath through the mouth, whilst humans prefer to breath through the nose and mouth. Therefore, lung deposition data is less relevant, and also, micron-sized particles may get trapped in the nasal–pharyngeal part of rodents; hence, lung profile may vary from that of human beings [208,209].

The respiratory tract has a very strong defensive mechanism, not only capable of pushing back the inhaled drug particles but also removing and inactivating their depots from the lungs. Therefore, the need for an ideal pulmonary DDS remains unmet and is badly affected by inadequate adherence due to mucociliary clearance and immune responses, along with the type of inhaler technique [210]. It is worth mentioning here that most of the investigations carried out to assess the protective effect of the antioxidant are based on murine and in vivo and/or clinical models and solely rely on dietary intake and/or systemic administration. Furthermore, the available literature also reflects that researchers explored only submerged cell lines and murine models without the aid of an inhalation technique, which overlooked many physiological aspects, too. Therefore, a more realistic model approach should be brought into practice so that the clinical transition can be more straightforward [211].

## 6. Conclusions

Although CD-based nano-sized drug delivery therapies focusing on lung deposition are still in the experimental phase, their potential in the areas of drug delivery, diagnosis, and gene knockdown cannot be ignored. Numerous derivatized CDs have been investigated for their physical, chemical, in vivo, and in vitro properties, which reveal that CDs can be utilized for antioxidant encapsulation in the management of oxidative stress-induced lung injuries. However, the concrete physiological testing model approach and inhalation devices are topics that warrant further exploration. Lastly, the biological fate of CDs and immunological response are also underexplored areas.

## Figures and Tables

**Table 1:** The exploitation of antioxidants in various lung diseases.

Disease	Antioxidants	Oxidative Stress Biomarker	In Vitro Model	In Vivo Model	Remarks	Ref.
PAH	Resveratrol	- SphK1-mediated NF-κB activation, - BMP/SMAD signaling pathway, - miR-638 and NR4A3/cyclin D1 pathway, - RhoA-ROCK signaling pathway.	--	Monocrotalin model in rats	- Resveratrol efficiently treated PAH via its action on signaling pathway, including interrupted proliferation of pulmonary arterial smooth muscle cells - Remodeling of right ventricle - The chemokine and cytokine-driven inflammatory response was reduced by controlling IL-6	[56]
BPD	Resveratrol	SIRT1 p53, Acetyl-p53 NLRP3 inflammasome IL-1β and IL-18 NF-κB		Neonate rats Model	- Assuaged hyperoxia-induced apoptosis in neonatal rats’ lung tissue by reducing ROS and p53. - Induced SIRT1 upregulation and - Declining of acetyl-p53 - Induced SIRT1 mRNA and protein expression	[47,57]
PAH	* Rhodiola crenulata * extract	PPARγ LC3B ATG7 p62 LKB1-AMPK	--	Rats with PAH	- The extract ameliorated PAH by inhibiting fatty acid oxidation and autophagy in PAH induced rats. - The extract also reversed the high level of decadienyl-L-carnitine by the modulation of metabolic enzyme CPT1A at mRNA and protein level in serum and lung in the rats having PAH	[58]
COPD	Fisetin	- Lowering NF-κB binding in the IL-8 promoter region in NCI-H292 lung epithelial cells, - Declined IL-8 release in response to TNF-α	Human lung epithelial cells NCI-H292 (CRL-1848) and HEK293T (CRL-3216) cells	--	- Effective in inhibiting the TNF-α/NF-κB signaling wordpathway by targeting protein kinase C. - Suppressed the inhibitor of IKK/NF-κB downstream signaling cascade	[59]
COPD	Resveratrol and genistein	NF-κB, TNF-α MMP-9	Isolated lymphocytes from healthy and COPD patients	--	- Increased level of TNF-α seen in MMP-9 and in COPD patients - Inhibitory mechanism against the translocation of NF-κB further reduces the concentration level of TNF-α and MMP	[60]
COPD	Crocin	NRF2	Assessment of cardiac parameters in COPD-induced rats by ECG under light anesthesia	Cigarette-induced lung injury model in rats	- Reduction in NRF2 - Cotinine concentration was increased - Protective effect against oxidative stress-induced lung injury - Cardiac abnormalities traced in electrocardiogram and hemodynamic parameters returned to normal range	[61]
LC	* Ilex latifolia * Thunb	Caspase-9 mRNA expression PI3K mTOR Bcl-2 NF-κB VEGF COX-2	Lung cancer A549 cell line Human normal lung epithelial cells BEAS-2B	--	- Conquer cancer by interrupting A549 cells’ proliferation, followed by apoptosis - BEAS-2B proliferation remained unaffected - Increased the levels of lactate dehydrogenase and ROS, hence promoting apoptosis - The presence of rutin, kaempferol, isochlorogenic acid A, isochlorogenic acid B, and isochlorogenic acid C was confirmed by HPLC analyses	[62]
Asthma	Luteolin	IgE IFN-γ IL-4 IL-5 BALF	--	Mouse model (BALB/c male mice)	- Promisingly decreased the threshold of antigen-induced bronchoconstriction - Reduction in IgE and IL-4/IL-5 concentration - Significant increase IFN-γ, level in BALF	[63]
CF	Olive Leaf Extract	CFTR F508del-CFTR	CFBE41o- HNE	--	- Avoided redox imbalance and inflammation - Declining in *P. aeruginosa* functioning - Synergism between extract and CFTR modulators has been established and can be employed as combinational therapy	[64]

BPD: Bronchopulmonary dysplasia, PAH: Pulmonary Arterial Hypertension, TNF: Tumor Necrosis Factor, NF-κB: Nuclear Factor-κB, MMP-9: Matrix Metalloproteinase-9, IgE: Immunoglobulin E, IFN-γ: Interferon Gamma, IL-Interleukin, BALF: Broncho-Alveolar Lavage Fluid, NRF2: Nuclear erythroid-related factor 2, PPARγ: Peroxisome Proliferator–activated Receptor γ, LKB1: Liver kinase B1, AMPK: Adenosine Monophosphate-activated Protein Kinase, ATG7: Autophagy Related Protein 7, LC3B: Microtubule-associated proteins light chain 3B, PI3K: Phosphatidylinositol 3-kinase, SIRT-1: Sirtuin-1, mTOR: Mammalian target of rapamycin, Bcl-2: B-cell lymphoma-2, VEGF: Vascular endothelial growth factor, COX-2: Cyclooxygenase-2, CFTR: Cystic fibrosis transmembrane conductance regulator, CFBE: CFBE41o- Human Cystic Fibrosis Bronchial Epithelial Cell, IKK/NF κB: Inhibitor of kappa B kinase.

**Table 2:** Overview of advantages and challenges of pulmonary delivery of nano-scale systems to treat lung diseases.

Advantages	Challenges	Ref.
Local or systemic lung treatment by delivering different drugs, such as natural, synthetic, macromolecules, DNA, proteins, peptides	Limited uptake and retention due to lung defense mechanisms, governed by ciliary clearance, macrophage phagocytosis, presence of mucus, and pulmonary surfactants	[94,95,98,99,108]
Enhanced solubility and bioavailability of hydrophilic compounds	Difficult targeting and undesired uptake	[95,96,97]
Size and surface modification using charged surfactants	Superficial deposition and high toxicity of cationic nanosystems	[107,109,110]
Avoidance of first-pass metabolism, sustained drug release, and reduced drug doses	Rapid clearance and immune responses onset	[89,90]
FDA-approved nano-formulations for lung cancer treatment (e.g., Abraxane^®^, Pazenirl^®^)	Difficult tumor site targeting and early drug loss due to reticuloendothelial system (RES) clearance	[111,117]

**Table 3:** Case studies of CD-based encapsulation of natural moieties.

Natural	Type of CDs	Target	Remarks	Ref.
Aurisin A	- β-CDs - (2,6-di-*O*-methyl-β-CDs) - HP-β-CDs	Anti-proliferative Activity against lung cancer cells	- Molecular dynamics simulations revealed that the stability and compactness of drug drug-conjugated complex were higher. - The introduction of drug is even responsible for enhancing the complex stability. - Upon encapsulation, the anti-proliferative activity of extract against A549 and H1975 lung cancer cells was significantly improved - High thermal stability achieved	[160]
Quercetin	- (2,6-di-*O*-methyl-β-CDs) - HP-β-CDs	Oxidative stress	- Both CDs have enhanced the pharmacological profile of Quercetin - In silico molecular dynamics confirmed various hydrogen bindings, supporting that both complexes can host quercetin - Calculations reflect that absolute binding free energies show that quercetin binds favorably to both CDs - Fluorescence spectroscopy shows moderate binding of quercetin in both CDs-based formulations	[159]
Olive leaf extracts and dexamethasone	- Thiolated HP-β-CDs	Oxidative stress in lungs	- The complex has promoted drug absorption while stabilizing nanoparticles against oxidative stress. - Promising for lung delivery, principally as stabilized nanoaggregates, proposing versatile administration for labile molecules like natural extracts.	[136]
Resveratrol	- SBE-β-CDs	Non-small Cell Lung Cancer	- Adequate drug deposition within lungs after nebulization of CDs based system - Enhanced the stability of resveratrol under physiological conditions - Significant cytotoxic effect was observed on five different Non-small Cell Lung Cancer cell lines	[129]
Resveratrol and Polydatin	- Polyvinylpyrrolidone/HP-β-CDs	Antioxidant and anti-inflammatory	- Immediate release of incorporated therapeutics - Penetration coefficients of both extracted molecules also increased - Antioxidant and anti-inflammatory activity against *P. cuspidati* extract was confirmed	[161]
Rosmarinic acid and Carvacrol.	- Maltodextrin	- Antioxidant, Anti-bacterial, Antifungal activities	- Encapsulation efficiency is more than 95% for both compounds of interest - Increased in vitro release of both the active compound	[162]
Curcumin	- α-, β-, and γ-CDs Polyethyleneglycol-Conjugated Gold Multifunctional Nanoparticles	Anticancer effect	- Amino-CDs conjugated with gold nanoparticles have been explored to achieve higher therapeutic effect of curcumin - Solubility of curcumin was improved, and remarkable high encapsulation efficiency was achieved - Proven in vitro cytotoxic effects on A549 cells were obtained	[163]
Hyperoside	- β-CDs - (2,6-di-O-methyl-β-CDs) - HP-β-CDs	Anti-microbial and anti-inflammatory effects	- Solubility was enhanced by 9-fold in the case of 2-hydroxypropyl-β-CD - Higher antioxidant activities in vitro and the H2O2–RAW264.7 cell model	[164]
*Boswellia carterii*	- HP-β-CDs - Epichlorohydrin-β-CDs	Inflammation	- The synergic effect of extract and formulation has been recorded - Reduction in level of ICAM-1, LTB4, and ILβ 4 - Improved histopathologic profiles	[165]
Tetrandrine	- HP-β-CDs	Pulmonary fibrosis	- Alleviated inflammation and pulmonary fibrosis - Regulated protein expression in the development of pulmonary fibrosis - Improved post-operative survival. - Drug delivered via nebulization was chiefly found to be located in the lungs only; hence, more local effect was seen	[139]
Rutin	- HP-β-CDs	Dietary supplementation	- Greater encapsulation efficiency and stability of rutin, at least for 30 days - Rutin release approaches the plateau around the sixth hour - No toxicity was observed in HepG2 cells - Significant increase in glucose uptake	[126]
Ligustrazine	- ROS-sensitive cross-linked covalent CDs framework	ALI	- Excellent aerodynamic properties, along with prominent antioxidant and anti-inflammatory capacities - Alleviated the inflammation, oxidant stress, and lung damage in rat models - Shielded the lungs by regulating the NRF2/NF-κB signaling pathway	[16]

## Data Availability

No new data were created.

## Abbreviations

The following abbreviations are used in this manuscript:

ROS	Reactive oxygen species
RNS	Reactive nitrogen species
IL-6	Interleukin 6
CS	Cystic Fibrosis
CFTR	Cystic Fibrosis Transmembrane Conductance Regulator
ALI	Acute Lung Injury
LC	Lung cancer
PH	Pulmonary Hypertension
COPD	Chronic Obstructive Pulmonary Disease
DDS	Drug Delivery System
CDs	Cyclodextrins
QS	Quorum Sensing
GelMA	Gelatin Methacrylate
BPD	Bronchopulmonary dysplasia
